# Analysis of Genetic and Non-genetic Predictors of Levodopa Induced Dyskinesia in Parkinson’s Disease

**DOI:** 10.3389/fphar.2021.640603

**Published:** 2021-04-29

**Authors:** Alfonsina Tirozzi, Nicola Modugno, Nicole Piera Palomba, Rosangela Ferese, Alessia Lombardi, Enrica Olivola, Alessandro Gialluisi, Teresa Esposito

**Affiliations:** ^1^ IRCCS Neuromed, Pozzilli, Italy; ^2^ Institute of Genetics and Biophysics, CNR, Naples, Italy

**Keywords:** SNCA, α-synuclein, levodopa induced dyskinesia, Parkinson disease, rs356219, D4S3481

## Abstract

**Background:** Levodopa (L-Dopa), representing the therapeutic gold standard for the treatment of Parkinson disease (PD), is associated with side effects like L-Dopa induced dyskinesia (LID). Although several non-genetic and genetic factors have been investigated for association with LID risk, contrasting results were reported and its genetic basis remain largely unexplored.

**Methods:** In an Italian PD cohort (N = 460), we first performed stepwise multivariable Cox Proportional Hazard regressions modeling LID risk as a function of gender, PD familiarity, clinical subtype, weight, age-at-onset (AAO) and years-of-disease (YOD), L-Dopa dosage, severity scores, and scales assessing motor (UPDRS-III), cognitive (MoCA), and non-motor symptoms (NMS). Then we enriched the resulting model testing two variants—rs356219 and D4S3481—increasing the expression of the *SNCA* gene, previously suggested as a potential mechanism of LID onset. To account for more complex (non-linear) relations of these variables with LID risk, we built a survival random forest (SRF) algorithm including all the covariates mentioned above.

**Results:** Among tested variables (N = 460 case-complete, 211 LID events; total follow-up 31,361 person-months, median 61 months), disease duration showed significant association (*p* < 0.005), with 6 (3–8)% decrease of LID risk per additional YOD. Other nominally significant associations were observed for gender—with women showing a 39 (5–82)% higher risk of LID—and AAO, with 2 (0.3–3)% decrease of risk for each year increase of PD onset. The SRF algorithm confirmed YOD as the most prominent feature influencing LID risk, with a variable importance of about 8% in the model. In genetic models, no statistically significant effects on incident LID risk was observed.

**Conclusions:** This evidence supports a protective effect of late PD onset and gender (men) against LID risk and suggests a new independent protective factor, YOD. Moreover, it underlines the importance of personalized therapeutic protocols for PD patients in the future.

## Introduction

Dyskinesia is characterized by involuntary dystonic and/or choreic movements of the trunk, limbs, and face (Finlay et al., 2014). One of the main risk factors predisposing to the onset of dyskinesia is Levodopa (L-Dopa) dosage, which currently represents the therapeutic gold standard for Parkinson’s disease (PD) (Coelho and Ferreira, 2014), which led to define a specific subtype of motor complications called L-Dopa Induced Dyskinesia (LID). Different risk factors have been associated with the onset of LID in PD patients, both modifiable and non-modifiable (Prashanth et al., 2011). Non-modifiable risk factors include age, gender, PD age at onset (AAO), clinical subtype, and genetic factors, while modifiable factors include L-Dopa dosage, duration of treatment, and body weight (Arabia et al., 2002; Sharma et al., 2006; Warren et al., 2013). Women generally show a greater incidence of dyskinesia than men (Zappia et al., 2005; Warren et al., 2013) and tend to develop dyskinesia earlier in relation to time of L-Dopa administration (Hassin-Baer et al., 2011). This may be due to a higher bio-availability of L-Dopa in women, which may be in turn be due to their usually lower body weight (Coelho and Ferreira, 2014). However, a higher LID risk for women has not been confirmed in other studies (Coelho and Ferreira, 2014; Campêlo and Silva, 2017). Similarly, early PD onset was associated with an increased risk of LID: a 5-years follow-up study of PD patients showed a prevalence of LID up to 50% at age 40–59, and of 16% after 70 years (Kumar et al., 2005), while another study found that the rates of dyskinesia in PD patients after 5 years of L-Dopa treatment were 70, 42, 33, and 24% for onsets at 40–49, 50–59, 60–69, and 70–79 years, respectively (Ku and Glass, 2010). Similarly, patients with AAO <40 years had a higher incidence of LID than those with AAO ≥50 years (Kostic et al., 1991). In line with this evidence, patients with longer disease duration—which is partly dependent on AAO—are more likely to develop LID (Ahlskog and Muenter, 2001). Of note, age has been detected as a risk factor in a single cross-sectional study, which reported a positive correlation between patients’ age and time-to-onset of motor complications, indicating that older patients develop dyskinesia later than younger patients (Schrag and Quinn, 2000). Similarly, disease duration has been also associated with prevalent risk of LID and motor fluctuations in a PD patients cohort from Ghana (Cilia et al., 2014). Among clinical subtypes of PD, the tremor-dominant subgroup has shown lower risk of dyskinesia compared to the bradykinetic and mixed manifestations subgroups (Zhang et al., 2013). PD stage has also been associated with LID risk, with patients in the early stage of the disease—with Hoehn and Yahr (HY) score of 1—showing a larger median time to LID from the beginning of L-Dopa treatment, compared to patients in a later HY stage (Kostic et al., 2002). Likewise, dyskinesia has been reported to be less frequent and less severe in late-stage PD patients (see Coelho and Ferreira, 2012 (Coelho and Ferreira, 2014)). A recent analysis of Chinese PD patients revealed a positive association of prevalent LID risk with high HY scores and low Unified Parkinson’s disease Rating Scale part III (UPDRS-III) under active L-Dopa treatment, which suggested progression of the disease and severity of motor symptoms as risk factors, in addition to early AAO, long disease duration, gender (women being more affected), and high L-Dopa equivalent dose (Zhou et al., 2019). Of note, the emergence of dyskinesia had no association with the initiation time of L-Dopa (Zhou et al., 2019). On the contrary, a community-based study found that the overall dose of L-Dopa was the most important predictor of motor fluctuations in PD patients, with dose and treatment having the strongest impact on LID prevalence (Schrag et al., 1998). These studies suggest that L-Dopa dosage may be more important than the duration of treatment. In other words, the higher the dose, the greater the risk of dyskinesia (Zhang et al., 2013).

Overall, PD patients show a remarkable heterogeneity in their response to L-Dopa and this likely suggests that there is a certain genetic predisposition toward the development of LID (Kalinderi et al., 2019). A handful of studies investigated so far variants in the genes encoding dopamine receptors (*DRD1*, *DRD2*, and *DRD3*), brain-derived neurotropic factor (*BDNF*) and leucine-rich repeat kinase 2 (*LRKK2*) (reviewed in (Kalinderi et al., 2019)), which often led to contrasting or inconclusive findings, warranting further genetic studies on LID. Another known PD-causative gene, *SNCA* (4q22.1, coding for α-synuclein) has been robustly implicated and investigated in PD etiology and progression (Stanic et al., 2016), but has so far been mostly neglected with regard to motor complications connected to the treatment of the disease, except for few studies. One of these identified a heterozygous autosomal dominant point mutation in the *SNCA* gene (c.158C > A; p. A53E in transcripts NM_000345.3, NM_001146054.1, NC_000004.11), in two Finnish related PD patients characterized by severe bradykinesia, very slight tremor and early onset of LID (Martikainen et al., 2015). In C. elegans models overexpressing human α-synuclein, chronic exposure to L-Dopa led to hyperactivity without meaningful increase in motor activity, this being proposed as a simple animal model of LID (Gupta et al., 2016). In spite of this interesting hypothesis, so far only two studies attempted to investigate the association between variants increasing *SNCA* expression levels and LID risk. Corrado et al. tested the influence of the 263 base pairs (bp) allele of the D4S3481 microsatellite marker on the incident risk of LID, in a longitudinal cohort of Italian PD patients (Corrado et al., 2018). They reported no significant differences between 263 allele carriers vs non carriers. More recently, Kim et al. (2020) tested an independent variant associated with the increase of *SNCA* expression levels—rs356219—in a longitudinal cohort of PD patients, reporting associations with motor fluctuations, but not with incident LID (Kim et al., 2020). Of note, these genetic studies were carried out in relatively small samples and are in contrast with preliminary evidence implicating α-synuclein levels in dyskinesia etiology (see above). This, along with the relatively comprehensive knowledge gained so far on *SNCA*, compared to other candidate genes, prompted us to focus on *SNCA* to further investigate potential genetic influences on LID onset (see below).

To sum up, research on risk and protective factors for LID onset present two main open issues. One is the partially contrasting results reported for many potential predictors by the different epidemiological studies, which warrant further investigations on the effect of these risk factors in cohorts of PD patients, possibly in a multivariable setting and across different ethnicities and ancestries, as well as within non-linear models. The other one is represented by the contrast among genetic/molecular studies involving *SNCA* (see above), which warrant further investigation of its influence on LID onset, especially of those variants leading to overexpression of the *SNCA* gene, to verify the hypothesis suggested by Gupta et al. (Gupta et al., 2016), or alternatively replicate the lack of associations detected for D4S3481 (Corrado et al., 2018) and rs356219 (Kim et al., 2020). Here, we investigated these two aspects, in a well characterized PD cohort from Central-Southern Italy (Gialluisi et al., 2019). First, we performed a multivariable survival analysis to analyze potential risk/protective effects of a number of non-genetic factors influencing LID risk. Then, we investigated in a similar setting potential effects of two variants previously implicated in PD etiology (Campêlo and Silva, 2017) and increasing the expression levels of *SNCA* gene, namely rs356219 (Fuchs et al., 2008; Pihlstrøm et al., 2018; Luo et al., 2019) and D4S3481 (Chiba-Falek et al., 2005; Fuchs et al., 2008; Trotta et al., 2012). Finally, we explored more complex relationships deploying exploratory supervised machine learning algorithms to detect the most influential variables on incident LID risk. We gained interesting insights in the epidemiology of LID and provide a notable contribution to knowledge in the field.

## Materials and Methods

### PD Patients Cohort and Outcome Definition

472 PD patients (288 males) from Central and Southern Italy were recruited at IRCCS Neuromed, Italy (Gialluisi et al., 2019). All the cases were diagnosed with PD according to published diagnostic criteria (Postuma et al., 2015). The project was approved by the institutional Ethical Committee and written informed consent was obtained from all the participating subjects. All analyses were carried out in R (see URLs).

The main outcome of survival analyses (absence/presence of dyskinesia) was assessed by a trained neurologist. The time-to-event was defined as the number of months between the beginning of L-Dopa treatment (based on consultation of the hospital database or self-report by the patients through drug prescription) and the onset of LID, or alternatively the end of follow-up time (right-censoring on December 31st 2018).

### Analysis of Incident LID Risk vs Non-genetic Factors

Basic assumptions of Cox Proportional Hazard (PH) regressions—proportionality of hazards, absence of outlier observations and linearity of effects—where checked for all the variables analyzed (see below), through Df-beta, Martingale and Schoenfeld residuals, where applicable.

To investigate the relation of non-genetic factors with the incident risk of LID, we applied a multivariable Cox PH regression—through the *cox. zph*() function of the *survival* package (see URLs) (Chung et al., 2014)—modeling LID onset as a function of gender, PD familiarity (familial/sporadic), clinical subtype (rigid-bradykinetic/tremorigenic/mixed), weight, PD AAO and years of disease (YOD), daily L-Dopa dosage (mg/day), HY scores, and scales assessing motor (UPDRS-III), cognitive (Montreal Cognitive Assessment, MoCA), and non-motor symptoms (NMS). Missing observations were imputed in all those patients with at least 50% of the measures available (N = 460), through a k-nearest neighbor (knn) algorithm implemented by the *kNN*() function (k = 10) of the *VIM* package (Zhang, 2015). Indeed, imputation is advised in presence of a patchy missing data pattern for variables with <50% missing values. This way, sample size was maximized to N = 460 (Table 1), which underwent statistical analyses.

**TABLE 1:** Description of the neuromed PD cohort used for analysis.

Set	Total	Fpd	Spd
N	460	196	264
Age (mean ± SD)	66.57 ± 8.84	66.31 ± 9.10	66.77 ± 8.65
AAO (mean ± SD)	58.17 ± 10.05	57.82 ± 10.55	58.42 ± 9.67
Disease duration (mean ± SD)	8.39 ± 6.16	8.51 ± 6.67	8.30 ± 5.77
Sex ratio (M/F)	282/178	124/72	158/106
Dyskinesia (D/ND)	211/249	98/98	113/151
PD clinical subtype (RB/t/mixed)	316/71/73	141/24/31	175/47/42
Weight (mean ± SD)	74.22 ± 10.92	73.47 ± 10.93	74.78 ± 10.89
BMI (mean ± SD)	26.90 ± 3.36	26.71 ± 3.46	27.05 ± 3.28
UPDRS (mean ± SD)	21.77 ± 10.44	21.09 ± 11.03	22.25 ± 9.98
MoCA (mean ± SD)	23.23 ± 4.72	23.19 ± 4.50	23.27 ± 5.03
HY (mean ± SD)	1.94 ± 0.87	1.92 ± 0.92	1.94 ± 0.83
NMS (mean ± SD)	56.39 ± 34.65	57.56 ± 34.95	55.52 ± 34.47

Here we report a description of PD cohort. AAO, age at onset; BMI, body mass index; D/ND, dyskinetic/non dyskinetic; HY, hoehn e yahr; M/F, male/female; MoCA, montreal cognitive assessment; NMS, non motor symptoms; RB/T, rigid-bradykinetic/tremorigen; SD, standard deviation; UPDRS, unified parkinson’s disease rating scale.

In Cox PH models, we implemented a multivariable stepwise regression approach through the *stepAIC*() function of the *MASS* package (see URLs), which kept within each model only those covariates determining a gain in the tradeoff between the goodness-of-fit of a given model and its parsimony (i.e., the number of parameters included) (Akaike, 2011). In other words, in this analysis only those covariates which significantly contributed to an increase in the total variance explained by the model—in spite of the addition of a parameter to the regression—were kept, allowing to “clean” the model for potential bias introduced by other collinear covariates. For this analysis we set a significance threshold *α* = 4.5 × 10^−3^, applying a Bonferroni correction for eleven different covariates tested. Moreover, full Cox regression models including all the covariates were run to compare results with stepwise regressions.

### Analysis of Incident LID Risk vs Candidate SNCA Variants

After investigating non-genetic variables, we analyzed the effect on LID onset of two *SNCA* genetic variants increasing the expression levels of the gene, namely rs356219 (Fuchs et al., 2008; Pihlstrøm et al., 2018; Luo et al., 2019) and D4S3481 (Chiba-Falek et al., 2005; Fuchs et al., 2008; Trotta et al., 2012) (see below for details on genotyping). To this end, we built multivariable Cox PH regression models adjusted for the non-genetic variables retained in the stepwise regression above. This analysis was driven by the hypothesis that these markers could affect LID onset, as previously suggested for other *SNCA* variants (see Introduction section). Specifically, we tested these associations under the assumptions of additive effect for the SNP rs356219, as previously tested by Kim et al. (Kim et al., 2020), and of a dominant effect for the microsatellite marker D4S3481, where carriers of the 263 bp allele (see below) were tested against non-carriers, as in Corrado et al. (Corrado et al., 2018). A Bonferroni correction for two independent genetic variants tested was applied, resulting in *α* = 0.025.

### Genotyping of the Candidate SNCA Variants

#### rs356219

rs356219 (hg19 coordinates chr4:90637601; A/G; allelic frequencies ∼49/51%)–lying in the 3′ untranslated region (3′UTR) of the *SNCA* gene (4q22.1) and previously associated with its circulating levels of expression (Chiba-Falek, 2001; Chiba-Falek et al., 2005; Fuchs et al., 2008; Cronin et al., 2009; McCarthy et al., 2011; Trotta et al., 2012; Cardo et al., 2014; Campêlo and Silva, 2017)–was genotyped using TaqMan® custom assays (Bio-Rad, United States), according to the manufacturer’s protocol, and analyzed in a Bio-Rad® CFX96™ Real Time PCR detection system. About 10–50 ng of DNA were amplified with 5 μL of 2X TaqMan universal PCR master mix, 0.5 μL of 20X primer and TaqMan probe dye mix. Cycling conditions were 3 min at 95°C, followed by 40 cycles of 15 s at 95°C and 30 s at 60°C. Genotyping was performed on 470 PD cases for which DNA samples were available at the time of genetic analyses.

We performed a general quality control (QC) of genotyped samples, in PLINK v1.9 (Cardo et al., 2014). These showed a call rate >98% (17 samples with missing genotype) and were in Hardy Weinberg Equilibrium (HWE, *p* = 0.62), suggesting a good quality of genotyping.

#### D4S3481 (Rep1)

D4S3481 (also known as Rep1) was analyzed in 469 PD patients of the Neuromed cohort, as described in (Maraganore et al., 1996) and in following studies (Corrado et al., 2018). Briefly, the region was amplified through polymerase Chain Reaction (PCR) from genomic DNA, using the following primer pairs: Fam5′-CCTGGCATATTTGATTGCAA-3′ and 5′-GAC​TGG​CCC​AAG​ATT​AAC​CA-3′. PCR reactions (25 μL final volume) containing 2 mmol/L MgCl2, 0.5 μM of each primer, 200 μM dNTPs, 1 unit of Taq polymerase (Life Technologies) and approximately 20 ng of genomic DNA. Thermal cycling was performed with an initial denaturation of 180 s at 94°C, followed by 35 cycles of 45 s at 94°C, 45 s at 61°C, 45 s at 72°C, and a terminal extension of 10 min at 72°C. PCR products were resolved by capillary electrophoresis on an ABI-3130XL DNA Analyzer (Applied Biosystem, Foster City, CA, United States), using GeenScan-500 ROX (Applied Biosystem) as molecular weight marker. Allelic sizes were assessed using the GeneMapper® Software Version 4.0 SNPlex™ (Applied Biosystem, Foster City, CA, United States). This method allows to determine the length of dinucleotide repeats at the investigated locus, and typically results in number of repeats ranging between 255 and 263. Since we detected only one sample carrying the 255 allele, and two samples with the 257 allele, and these alleles are usually neglected due to their low frequency (Corrado et al., 2018), we removed them before the analyses, as done elsewhere (Maraganore et al., 1996). This variant showed genotyping call rate >97% (29 samples with missing genotype) and was in HWE (*p* = 0.28).

### Machine Learning Analyses

To validate observations made in Cox PH models and explore more complex relationships of the investigated predictors with LID risk, we built survival random forest (SRF) algorithms using the above mentioned covariates as input features and dyskinesia events and time-to-dyskinesia as a label. SRF represent a class of supervised machine learning algorithms used to predict incident outcomes in a longitudinal setting and are potentially more powerful than classical statistical models since they model also non-linear and interactive functions in the prediction. In particular, two models were deployed, both including and excluding *SNCA* variants as features. SRF were built using the *rfsrc*() function of the randomForestSRC package in R (see URLs). After hyperparameter tuning, model training and testing, we performed a variable importance analysis (*vimp*() function) to establish the most important features (or variables) influencing the prediction of incident LID events. Details on the construction of the SRF are reported in Supplementary Methods.

## Results

### Survival Models With Non-genetic Predictors

The multivariable Cox PH regression analyzing the association of non-genetic factors with the incident risk of LIDs was performed in 460 PD cases for which all phenotypic, clinical and pharmacological information was available after imputation, with a total of 211 LID events. These subjects were followed for a total of 31,361 person-months (median follow-up time 61 months). The AIC-based stepwise approach retained five variables in the model, namely gender, PD familiarity, age-at-onset (AAO), staging (H&Y) and duration (YOD), whose associations with the incident risk of LID are reported in Table 2. In particular, YOD showed significant associations surviving correction for multiple testing, with a longer duration of disease being protective against LID onset (HR [CI] per additional year of disease = 0.94 [0.92–0.97], *p* = 1.4 × 10^−4^). Nominally significant associations (*p* = 0.02) were observed for AAO (HR [CI] = 0.98 [0.97–1.00] per year increase) and gender (HR [CI] = 1.39 [1.05–1.82] for women compared to men), although they did not survive Bonferroni correction (see Table 2).

**TABLE 2:** Results of the stepwise multivariable Cox PH regression modeling LID onset vs non-genetic factors.

Exposure	HR	CI (lower)	CI (upper)	z	p
Gender (women)	1.39	1.05	1.82	2.33	0.02
Familiarity	1.24	0.95	1.63	1.55	0.12
AAO	0.98	0.97	1.00	−2.33	0.02
Hoehn and yahr	0.89	0.77	1.04	−1.43	0.15
YOD	0.94	0.92	0.97	−3.80	1.4 × 10^−4^

Here we report only exposures which were retained in stepwise multivariable models. AAO, age at onset; CI (lower/upper), inferior and superior limits of the 95% Confidence Interval of HRs; HR, hazard ratio; *p*, *p*-value; YOD, years of disease; z, z-score. Significant associations surviving Bonferroni correction (*p* < 4.5 × 10^−3^) are highlighted in bold.

### Exploratory Survival Models With Genetic Predictors

**FIGURE 1:**
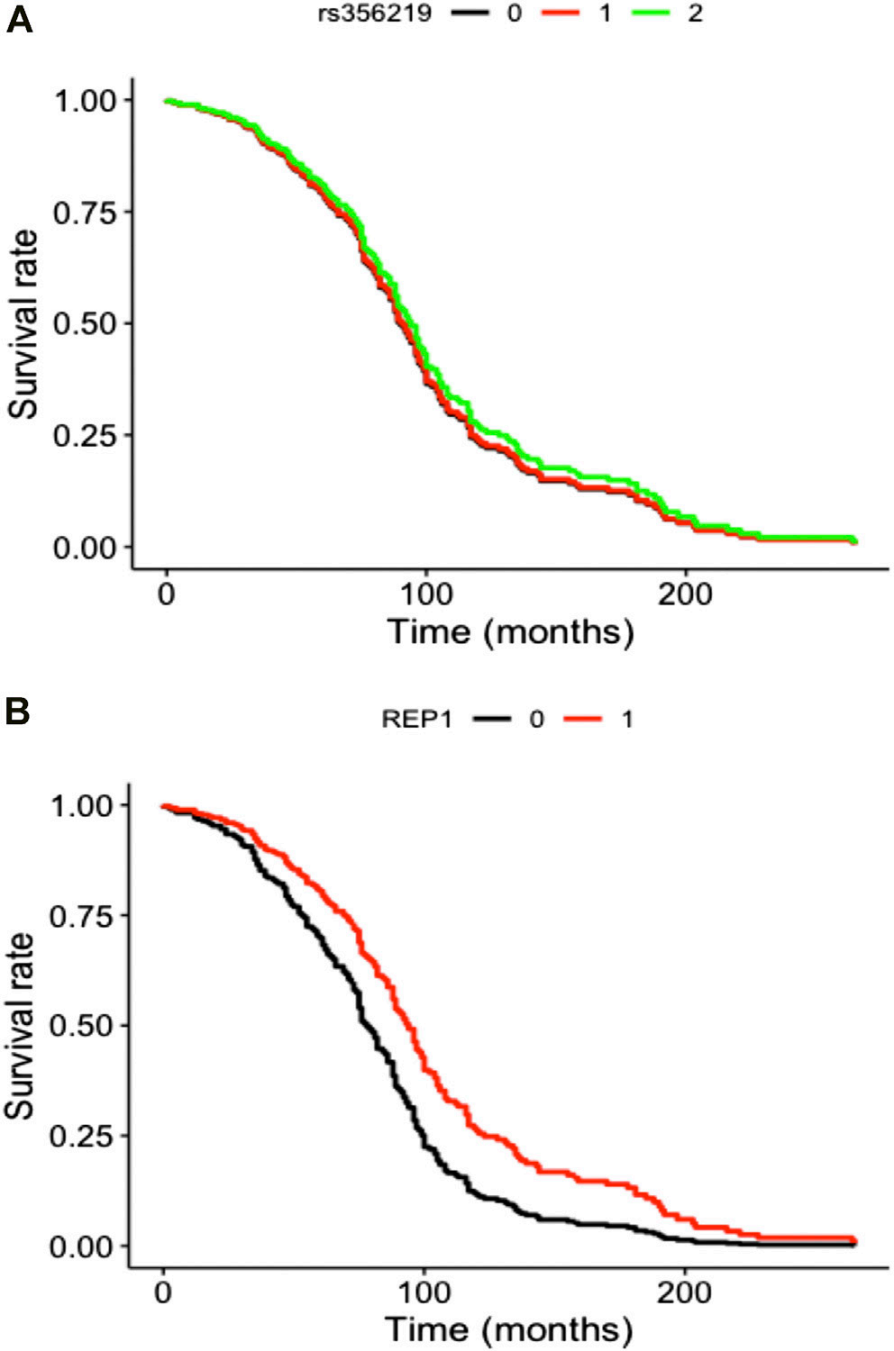
Cox curves of genetic model tested modeling incident LID risk vs **(A)** additive genetic model of rs356219 and vs **(B)** pseudo-recessive genetic model of D4S8134. Genotype classes were defined as **(A)** AA (black), AG (red) and GG (green) genotypes for rs356219 and **(B)** 263 bp allele carriers (black) vs all other genotypes (red) for D4S8134.

SNCA genetic variants were analyzed in a final sample size of N = 456 for rs356219 and N = 455 for D4S3481, with a total of 208 LID events for rs356219 and 210 LID events for D4S3481. Total follow-up time was 31,153 person-months for rs356219 and 31,140 for D4S3481, while median time was 61 months for rs356219 and 60.5 for D4S3481. We observed no statistically significant genetic effects of these variants on incident LID risk (Figures 1A,B), although D4S3481-263 allele carriers showed a nominally significant protective association with incident LID (Supplementary Table S2). This prompted us to compare follow-up times of the Rep1 263 bp allele carriers vs non-carriers through a rank sum test, which revealed a significant difference between the two groups (W = 4.18, *p* = 0.04), with 263 bp allele carriers showing a higher follow-up time (Supplementary Figure S1A), in line with the protective effect observed in the survival analysis (Supplementary Table S2). No differences were observed in the follow-up time of rs356219 genotype classes (Supplementary Figure S1B).

### Survival Random Forest

**FIGURE 2:**
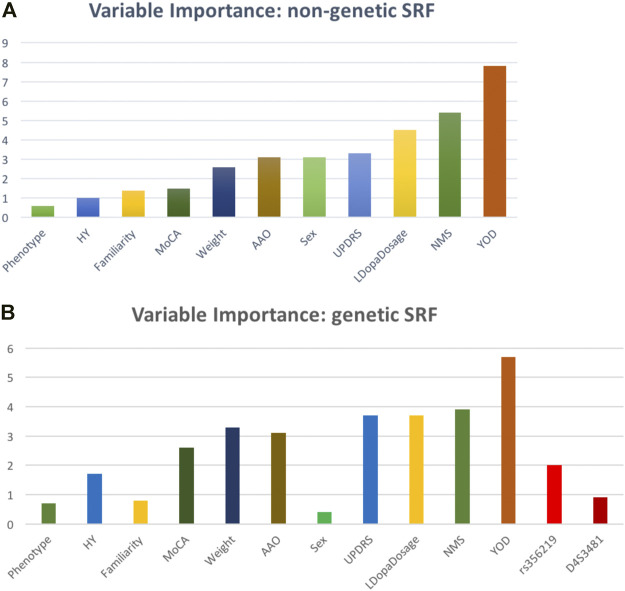
Relative variable importance (%) of the LID risk predictors tested within the survival random forest **(A)** excluding and **(B)** including genetic (*SNCA*) features.

A SRF model predicting LID onset as a function of non-genetic predictors showed a sensitivity of 39% and a specificity of 76% in the test set. A variable importance analysis (Figure 2A) revealed that the most influential features in predicting incident LID risk were YOD (standardized feature importance 7.8%), NMS (5.4%) and L-Dopa dosage (4.5%). In the genetic SRF (including also SNCA variants) performance was slightly worse (sensitivity: 27%, specificity: 73%), but the feature importance rank was substantially stable (YOD: 5.7%, NMS: 3.9%, L-Dopa dosage: 3.7%), with SNCA variants showing a negligible contribution (Figure 2B).

## Discussion

In the present study, we analyzed the influence of both non-genetic and genetic factors on the incident risk of LID in a cohort of Italian PD patients through a multifaceted approach implying the use of both classical statistics and supervised machine learning methods, which revealed two main findings.

First, a multivariable analysis of non-genetic exposures previously implicated in LID onset showed a significant association with years of disease (YOD), which in our study conferred a protective effect. This is in contrast with previous studies reporting that patients with longer disease duration—which is only partly dependent on age at onset (AAO)—are more likely to develop LID (Ahlskog and Muenter, 2001; Cilia et al., 2014; Coelho and Ferreira, 2014; Tran et al., 2018; Zhou et al., 2019). This discrepancy may be explained by the multivariable setting of our exploratory analysis, where also AAO showed a significant association with incident LID risk, in the expected direction (see below for further discussion). Second, the same multivariable models revealed a nominally significant association of incident LID risk with gender, in line with previous studies reporting a higher risk of LID for women compared to men (Zappia et al., 2005; Warren et al., 2013). However, this association was attenuated after constraining the model to adjust for weight (see full Cox PH model). This suggests that a generally lower weight of women and the resulting higher bioavailability of levodopa may play a role in this association (Coelho and Ferreira, 2014). We also observed a protective effect of late PD onset against LID risk. These findings corroborate previous observations reporting negative associations of LID risk with AAO (Kumar et al., 2005; Ku and Glass, 2010; Warren et al., 2013; Hashim et al., 2014). Importantly, these associations were observed in a multivariable setting and were all mutually independent, although only that with duration of disease survived a conservative correction for multiple testing and persisted mutual adjustments of all the other covariates in the full model.

Of note, we observed no evidence for a linear association between L-Dopa daily intake and incident LID risk, in spite of previous literature reporting it as one of the most important risk factors for dyskinesia (Warren et al., 2013; Coelho and Ferreira, 2014; Eusebi et al., 2018). This may be due to the fact that different studies analyzed differently exposure to L-Dopa intake. Indeed, some reported association with L-Dopa dosage at the beginning of the pharmacological treatment (Warren et al., 2013), while others took the latest prescription as dosage of reference (Bjornestad et al., 2016), and other works analyzed L-Dopa Equivalent Daily Dosage (LEDD) (Tomlinson et al., 2010; Zhou et al., 2019). Interestingly, our survival random forest model reported L-Dopa dosage among the most influential features, suggesting that it may contribute to LID risk in a more complex, non-linear fashion. While the topic is still controversial, previous pioneering studies in the field have previously reported a concordant evidence with our observation on L-Dopa. In a large African cohort of PD patients, Cilia and colleagues observed that motor fluctuations and dyskinesias were not associated with the duration of L-Dopa therapy, but rather with higher levodopa daily dose, beyond longer disease duration (Pandey and Srivanitchapoom, 2017). Others have reported the prevalence of LID to increase with disease and treatment duration, and that it usually takes approximately 3–5 years after administering L-Dopa for developing dyskinesia (Bjornestad et al., 2016). Our findings well fit the hypothesis that L-Dopa substantially increases the risk of LID, although this influence might dissipate in the long term (Coelho and Ferreira, 2014). Overall, these lines of evidence support the idea that the practice to delay L-Dopa therapy to postpone the occurrence of LID may not be justified for all PD patients. In the future, a personalized approach to L-Dopa therapy based on the characteristics of the patients may be more suited to minimize risk-to-benefit ratio. While we are still far from this goal and large and well characterized PD datasets are warranted to this purpose, this study represents a first exploratory attempt in this perspective.

When we examined candidate *SNCA* genetic variants, we observed only a nominally significant protective influence of 263 bp allele of D4S3481 against incident LID risk, compared to non-carriers in a dominant model, while no evidence of association for rs356219 was detected. Similarly, SRF models including also *SNCA* genetic variants revealed a negligible contribution to the prediction of incident LID risk. This evidence is in line with the lack of longitudinal associations with LID previously reported by Corrado et al. for D4S3481 (Corrado et al., 2018) and by Kim et al. for rs356219 (Kim et al., 2020). At present, it remains difficult to say whether this is due to the total lack of influence of these two variants—or possibly of the *SNCA* gene as a whole—on the occurrence of LID, since both have been under-investigated in this regard. Overall, further genetic studies on these and other *SNCA* variants are warranted in larger samples, to clarify the relation of this gene with LID onset and risk, which has been fairly neglected so far.

### Strengths and Limitations

In addition to the lack of detailed information on L-Dopa treatment duration for some PD patients, additional limitations which may have limited power include the relatively low sample size of the study, which was however counterbalanced by the use of advanced machine-learning based imputation techniques to maximize N. Computing L-Dopa equivalent daily dose (LEDD) for each participant may help to have a slightly more precise and comparable information to sum dopamine dosages coming from different sources (e.g., carbidopa). Although different approaches have been suggested to compute LEDD, no agreement has been reached on a gold standard procedure and different studies report different L-Dopa dosage exposures (Bjornestad et al., 2016; Zhou et al., 2019). Finally, the assessment of dyskinesia made by qualified neurologists only reported the absence/presence of motor complications, hence missing information on the time spent with or without LID in the different stages of the disease, as well as on the severity of motor complications. We are now planning a recall of the cohort to assess in detail these aspects.

In spite of these limitations, this still represents one of the largest genetic studies on LID and one of the most richly characterized as for non-genetic exposures. Indeed, to our knowledge no attempt has been made so far to predict LID onset through supervised machine learning techniques, which show a notable power compared to classical statistical models since they account also for more complex relationships of the investigated predictors and may pave the way to personalized medicine approaches.

Overall, we provide a contribution to knowledge on the epidemiology of LID, which will help to better understand both environmental and genetic influences of this phenomenon. This represents an important translational milestone in developing future personalized strategies for the treatment and management of PD patients in the future.

## Data Availability

The raw data supporting the conclusions of this article will be made available by the authors, without undue reservation, to any qualified researcher.
