# Supplementary material for: Analysis of Genetic and Non-genetic Predictors of Levodopa Induced Dyskinesia in Parkinson’s Disease
Source: Front Pharmacol. 2021 Apr 29;12:640603. doi: 10.3389/fphar.2021.640603 (PMC8118664; doi:10.3389/fphar.2021.640603)
Supplement: Supplementary file 1 [file datasheet1.docx]

Supplementary Material

**Survival Random Forest (SRF)**

To take into account potential more complex (e.g non-linear and interaction) influences of the investigated predictors on incident LID, we carried out an exploratory machine learning analysis to establish feature importance in the prediction of LID risk. To this end, we deployed two SRF algorithms trough the *rfsrc()* function of the randomForestSRC package in R (see URLs), one including all non-genetic predictors - namely gender, PD familiarity, phenotype, age at onset (AAO), years of disease (YOD), staging (Hoehn & Yahr score), motor (UPDRS-III), cognitive (MoCA score) and non-motor symptoms (NMS), last L-Dopa dosage and weight of each patient, hereafter called “non-genetic” SRF model – and one including also genetic variants tested (rs356219 and D4S3481), hereafter called “genetic” SRF model. The pipeline implied i) removal of collinear variables (e.g. age, BMI and height) and elaboration or remaining variables (min-max normalization for continuous scores); ii) hyperparameter tuning through the *train()* function of the caret package (see URLs) to optimize the performance of the algorithm (which was reached for nodesize = 9, mtry = 5, ntree = 100 in the non-genetic model and for nodesize = 9, mtry = 4, ntree = 100 in the genetic model); iii) training of the model in a random subset (70%) of the analyzed dataset; iv) testing in the remaining 30% of the dataset (test set), with evaluation of classification performance; v) feature importance analysis to determine the most influential variables within the SRF model, through the *vimp()* function.

**Table S1. Results of Cox PH regressions modelling incident LID risk vs non-genetic factors.**

|  | HR | CI (lower) | CI (upper) | z | p |
| --- | --- | --- | --- | --- | --- |
| Gender (woman) | 0.78 | 0.91 | 1.78 | 1.44 | 0.15 |
| Familiarity | 0.83 | 0.91 | 1.60 | 1.28 | 0.20 |
| Phenotype (tremorigenic) | 1.37 | 0.43 | 1.23 | -1.19 | 0.24 |
| Phenotype (mixed) | 1.12 | 0.61 | 1.32 | -0.55 | 0.58 |
| AAO | 1.02 | 0.97 | 1.00 | -2.02 | 0.04 |
| UPDRS | 1.01 | 0.98 | 1.01 | -0.76 | 0.44 |
| LDopaDosage | 1.00 | 1.00 | 1.00 | -0.10 | 0.92 |
| MoCA | 0.97 | 0.38 | 2.79 | 0.07 | 0.95 |
| HY | 1.12 | 0.76 | 1.06 | -1.31 | 0.19 |
| YOD | 1.06 | 0.92 | 0.98 | -3.44 | 6×10^-4^ |
| NMS | 1.00 | 1.00 | 1.01 | 0.46 | 0.65 |
| Weight | 1.00 | 0.98 | 1.01 | -0.56 | 0.57 |

Hazard Ratio (HR), relevant 95% Confidence Interval (CI), z-score (z) and p-value (p) are reported for the variable tested modelling incident LID risk vs non-genetic factors.

**Table S2. Results of Cox PH regressions modelling incident LID risk vs rs356219 and D4S3481.**

| Genetic Variant | Genetic Model | Contrasta | HR [CI] | z | p |
| --- | --- | --- | --- | --- | --- |
| rs356219 | Additive | AG vs AA | 0.92 [0.68; 1.25] | -0.52 | 0.61 |
|  |  | GG vs AA | 0.96 [0.65; 1.42] | 0.21 | 0.83 |
| D4S3481 | Pseudo-recessive | 263 allele carriers  vs all others | 0.60 [0.37; 0.98] | -2.04 | 0.041 |

Hazard Ratio (HR), relevant 95% Confidence Interval (CI), z-score (z) and p-value (p) are reported for the genetic variants tested, adjusted for sex, familiarity, age at onset, years of disease and Hoehn & Yahr score. None of these genetic models survived correction for multiple testing (α = 0.025).

**Figure S1. Boxplots of dyskinesia-free time for a) rs356219 and b) D4S3184 genotype classes tested in the main genetic model.**

a)


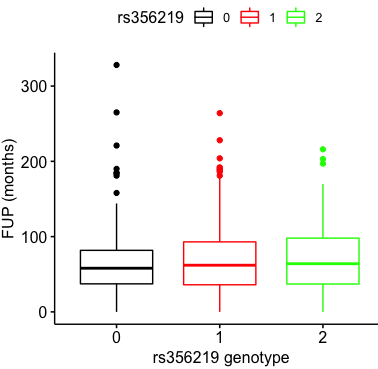


b)

**
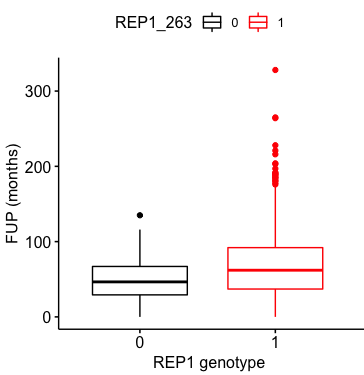
**

Here, median and interquartile range of time-to-dyskinesia – defined as follow-up time until LID event or censoring - by genotype class is reported, for the genetic models tested. Genotype classes compared were a) AA (black), AG (red) and GG (green) for rs356219 (Additive model) and b) 263 allele carriers (red) vs all other genotypes (black) for Rep1**
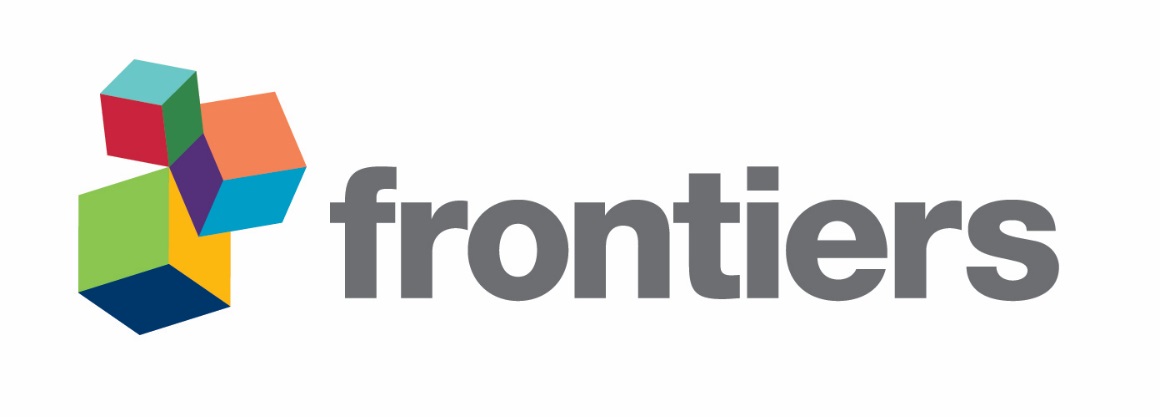
**
